# O-GlcNAc cycling in the developing, adult and geriatric brain

**DOI:** 10.1007/s10863-018-9760-1

**Published:** 2018-05-22

**Authors:** Olof Lagerlöf

**Affiliations:** Department of Neuroscience, Karolinska Institutet, 171 77 Stockholm, Sweden

## Abstract

Hundreds of proteins in the nervous system are modified by the monosaccharide O-GlcNAc. A single protein is often O-GlcNAcylated on several amino acids and the modification of a single site can play a crucial role for the function of the protein. Despite its complexity, only two enzymes add and remove O-GlcNAc from proteins, O-GlcNAc transferase (OGT) and O-GlcNAcase (OGA). Global and local regulation of these enzymes make it possible for O-GlcNAc to coordinate multiple cellular functions at the same time as regulating specific pathways independently from each other. If O-GlcNAcylation is disrupted, metabolic disorder or intellectual disability may ensue, depending on what neurons are affected. O-GlcNAc's promise as a clinical target for developing drugs against neurodegenerative diseases has been recognized for many years. Recent literature puts O-GlcNAc in the forefront among mechanisms that can help us better understand how neuronal circuits integrate diverse incoming stimuli such as fluctuations in nutrient supply, metabolic hormones, neuronal activity and cellular stress. Here the functions of O-GlcNAc in the nervous system are reviewed.

## Introduction

### Neuronal circuits drive behavior

Brain function relies on the concerted action of networks of neurons. Individual cells influence how thoughts and emotions occur in the mind. Some behaviors are disrupted completely if specific neurons are damaged. Nevertheless, almost no function of the nervous system depends solely on a single cell type. Neuronal circuits with different effects on behavior often lie intermingled and stretch over large parts of the brain. Manipulation of anatomical regions often does not lend enough specificity to understand how behaviors are encoded in the brain. However, the last decade has witnessed an explosion in available tools to interrogate defined neuronal circuits. Genetic identification of cells in particular has enabled mapping of distinct behaviors to discrete neuronal pathways. These studies emphasize that it is only by studying communication between synaptically or otherwise coupled cells that information processing in the brain may be understood (McCulloch and Pitts 1990; Denk et al. 2012; Huang and Zeng 2013; Sohn et al. 2013; Tovote et al. 2015; Tsien 2015).

### Protein function is regulated by O-GlcNAc

Connecting circuit structure with circuit function requires investigation of the molecular events by which participating cells respond to incoming stimuli and signal to each other (Kessels and Malinow 2009; O'Rourke et al. 2012; Shepherd and Huganir 2007). Until the early 1980's, it was known that protein function could be modified by attaching complex carbohydrates to mainly their asparagine, serine or threonine residues. This kind of glycosylation occurs in the cellular secretory pathway on proteins that are expressed topologically outside the cell. Then Gerald W. Hart's laboratory at Johns Hopkins University discovered that the monosaccharide D-N-acetylglucosamine (GlcNAc) is covalently coupled through an O-glycosidic bond (O-GlcNAc) to many proteins in the cytoplasm and nucleus (Torres and Hart 1984; Holt and Hart 1986). O-GlcNAc is attached in β-linkage to the hydroxyl group of serine and threonine amino acid side chains. Unlike classical protein glycosylation O-GlcNAc is rarely elongated and can cycle on and off proteins faster than the peptide backbone turns over and this on a time scale of minutes to hours (Roquemore et al. 1996; Yuzwa et al. 2008; Song et al. 2008; Chou et al. 1992).

O-GlcNAcylation of specific sites can affect, for example, peptide structure, enzyme activity, ion channel conductivity and protein-protein interactions (Chen et al. 2006; Dias et al. 2009; Ruan et al. 2014; Myers et al. 2016; Tarrant et al. 2012; Hart et al. 2011; Tarbet et al. 2018). In the nervous system, other important examples of O-GlcNAc function include protein stability and solubility (Yuzwa et al. 2012; Marotta et al. 2015). Today more than 4000 proteins modified by O-GlcNAc have been described (Ma and Hart 2014). These regulate numerous and diverse cellular events such as transcription, translation, signaling and ROS production in the mitochondria (Bond and Hanover 2013; Tan et al. 2017a). As we shall see, O-GlcNAc is particularly abundant in the brain where neuron-specific proteins and proteins common to most cells are O-GlcNAcylated.

### O-GlcNAcylation is regulated by OGT and OGA

The adding and removing of O-GlcNAc is controlled by only two enzymes. O-GlcNAc transferase (OGT) attaches and O-GlcNAcase (OGA) detaches O-GlcNAc from proteins. Both OGT and OGA are expressed in the nucleus, cytosol and mitochondria where they dynamically interact with the respective substrates (Kreppel et al. 1997; Haltiwanger et al. 1992; Dong and Hart 1994; Gao et al. 2001; Banerjee et al. 2015; Hart et al. 2011). There is an O-GlcNAc transferase called eOGT that has been identified in the secretory pathway, but this enzyme is distinct from OGT (Matsuura et al. 2008; Varshney and Stanley 2017).

Changes to the specific activity or expression levels of OGT or OGA can increase or decrease global O-GlcNAc levels (Hart et al. 2011). OGT's donor substrate is UDP-GlcNAc. UDP-GlcNAc is produced in the cell through the hexosamine biosynthesis pathway (HBP) (Hart et al. 2011). In adipocytes, the HBP converts about 2-3% of all glucose entering the cell to UDP-GlcNAc (Marshall et al. 1991). In addition to glucose, amino acid, fatty acid and nucleotide metabolism also feed into the HBP (Hart et al. 2011). The conversion rate is controlled by the glutamine:fructose-6-phosphate amidotransferase (GFAT). The affinity of GFAT for its substrate, fructose-6-phosphate, is relatively poor and if nutrient availability increases, so do UDP-GlcNAc levels (Bouche et al. 2004; Wang et al. 1998; Hawkins et al. 1997; Marshall et al. 2004). GFAT affects the downstream effects of glucose over a wide range of glucose concentrations (Sayeski and Kudlow 1996). The regulation of the HBP is complex however and whether diabetic glucose concentrations transduce into similar fluctuations of cellular UDP-GlcNAc levels has been challenged in adipocytes (Bosch et al. 2004; Schleicher and Weigert 2000). Nonetheless, the activity of OGT is sensitive to altered UDP-GlcNAc levels from nano- to millimolar concentrations (Slawson et al. 2010). Raised UDP-GlcNAc levels increase OGT activity against peptides and proteins *in vitro* (Shen et al. 2012; Kreppel and Hart 1999). Numerous reports show also that overall O-GlcNAcylation depends on the cell's access to nutrients in multiple *ex vivo* culture systems and in organisms. We shall see in forthcoming sections that though the metabolic control of O-GlcNAc is complex, OGT is today recognized as a central and global energy sensor in the cell due to nutrient-dependent flux through the HBP (Slawson et al. 2010).

In reverse, complete deprivation of glucose can stimulate global O-GlcNAc levels dramatically (Cheung and Hart 2008; Taylor et al. 2008). As first shown by Zachara *et al*, O-GlcNAc, in fact, responds to and regulates the cell's capacity to handle multiple forms of stress (Zachara et al. 2004). The mechanism is multifaceted and still under investigation but stress-induced O-GlcNAcylation results under some conditions from a higher total OGT activity because of altered flux through the HBP or transcription of *Ogt* (Wang et al. 2014a; Cheung and Hart 2008; Taylor et al. 2008).

The proteins that become O-GlcNAcylated during stress are at least partly different than the ones being modified during eu- or hyperglycemia (Taylor et al. 2008; Groves et al. 2017). Other stimuli can lead to highly compartmentalized regulation of O-GlcNAc. O-GlcNAcylation is sometimes promoted, depressed or unaffected in different parts of the cell simultaneously (Kearse and Hart 1991; Carrillo et al. 2011; Griffith and Schmitz 1999; Yang et al. 2008). If and when a particular protein is O-GlcNAc modified is thought to depend to a large extent on the context in which OGT and OGA operate. OGT and OGA form complexes with a large set of proteins and these binding-partners can direct O-GlcNAcylation towards specific targets (Hart et al. 2011; Groves et al. 2017). Some interactors are associated with changes in OGT and OGA specific activity (Groves et al. 2017; Marz et al. 2006). Similarly, activity and substrate selectivity can be affected also by direct phosphorylation (Bullen et al. 2014; Whelan et al. 2008).

In addition to extrinsic regulation of substrate targeting, what proteins are O-GlcNAcylated at a given time and place depends on factors intrinsic to OGT and OGA. Surprisingly, whereas a raised UDP-GlcNAc concentration elevates O-GlcNAc on most targets, the relative increase of O-GlcNAc on peptides and proteins *in vitro* differs between substrates, suggesting that UDP-GlcNAc influences OGT's intrinsic specificity (Kreppel and Hart 1999; Shen et al. 2012). It should also be noted that whereas much evidence argues that there is no absolute consensus sequence for where O-GlcNAcylation occurs, the local peptide environment and neighboring amino acids do affect which serines or threonines are preferred (Hart et al. 2011; Nagel and Ball 2014). Plus, alternative splicing affects the subcellular expression of OGT and OGA, and possibly intrinsic substrate preference (Nagel and Ball 2014).

While O-GlcNAc levels can fluctuate globally within the cell, the idea of OGT and OGA as holoenzymes functioning in complexes with other proteins in dynamic multicellular environments opens up the possibility of local and substrate-specific regulation of O-GlcNAcylation (Lagerlof and Hart 2014; Hart et al. 2011). One way of synthesizing global and local regulation would be to understand them as operating simultaneously but on separate levels. There are, however, many aspects of OGT and OGA function that still are not understood. It has been shown, for example, that OGT can act as a protease against at least one substrate (host cell factor 1) and may be important as a scaffold for other proteins, in addition to its ability to O-GlcNAcylate proteins (Levine and Walker 2016).

### O-GlcNAc, brain function and disease

How only two enzymes coordinate via O-GlcNAc the function of thousands of proteins has only begun to be explored. If dysregulated, aberrant O-GlcNAcylation can lead to disease. It will be described below that the genes encoding OGT and OGA are linked to late-onset diabetes, intellectual disability and several neurodegenerative disorders. OGT and OGA function in homeostasis where too high or too low O-GlcNAc levels can deter cellular physiology (Yang and Qian 2017; Zhang et al. 2014; Bond and Hanover 2013). O-GlcNAc is a nutrient and stress sensor in the nervous system. The cellular identity and the extended morphology of neurons and glia, however, determine the response and functional consequence of O-GlcNAc to common and neuron-specific stimuli. Specific neuronal circuits drive behavior and it is only from their perspective the function of O-GlcNAc can be understood. There are many extensive reviews on O-GlcNAc function in general (Hart et al. 2011; Ruan et al. 2013; Bond and Hanover 2013; Hardiville and Hart 2014; Levine and Walker 2016; Yang and Qian 2017; Nagel and Ball 2014). Here major roles played by O-GlcNAc in the nervous system are discussed.

## O-GlcNAc and its regulation in the nervous system

### The O-GlcNAc modification in the nervous system

Almost all studies characterizing O-GlcNAc in the nervous system have focused on the forebrain and the cerebellum. Immunohistochemistry indicates that O-GlcNAc is comparatively more abundant under basal conditions in some brain regions and some cells within a given area (Liu et al. 2012; Rex-Mathes et al. 2001; Akimoto et al. 2003; Taylor et al. 2014; Lagerlof et al. 2016). No quantitative comparison has been made but authors have argued O-GlcNAc to be concentrated particularly in, for example, Purkinje cells in the cerebellum and under some conditions pyramidal neurons of the CA1 region of the hippocampus (Taylor et al. 2014; Liu et al. 2012; Akimoto et al. 2003; Rex-Mathes et al. 2001). The staining pattern in the hippocampus is broadly similar between rodents and humans (Taylor et al. 2014). Total O-GlcNAc in brain homogenate is the highest during pre- and perinatal development and decreases during later development (Yanagisawa and Yu 2009; Liu et al. 2012). *In vitro*, early neuronal differentiation also has been associated with a decrease in global O-GlcNAcylation, albeit the global O-GlcNAc levels appear to cycle from day to day (Andres et al. 2017; Speakman et al. 2014). At around one month after birth, overall O-GlcNAc plateaus at a relatively low level and then remains stable for most of adulthood (Liu et al. 2012). While some have argued that these levels (in rats) persist in the geriatric brain (Liu et al. 2012), others have shown an increase at similar ages in both mice and rats (Yang et al. 2012; Fulop et al. 2008). However, the pattern of O-GlcNAcylation using western blotting has been reported consistently to change in the brain across all stages of life, including in the very old animal (Yang et al. 2012; Liu et al. 2012; Fulop et al. 2008). Hence, the relative abundance of O-GlcNAc between proteins continuously fluctuates.

The proteins in the brain and the peripheral nervous system that are O-GlcNAcylated belong to a wide array of functional categories (Lagerlof and Hart 2014; Kim et al. 2016; Wang et al. 2017). Just like in other tissues, mass spectrometry has mapped O-GlcNAc sites on proteins involved in, for example, signaling, transcription, translation and cytoskeletal regulation (Alfaro et al. 2012; Wang et al. 2010a; Khidekel et al. 2004). While many of these proteins are shared with non-neuronal organs, some play specialized roles in neurons, e.g. the transcription factor cAMP responsive element-binding protein (CREB) (Altarejos and Montminy 2011; Feldman 2009; Rexach et al. 2012). Other O-GlcNAc proteins such as neurotransmitter receptors or some synaptic proteins are expressed mainly or only in the nervous system (Trinidad et al. 2012; Vosseller et al. 2006; Schoch et al. 1996). Immuno-electron microscopy and biochemical fractionation coupled with western blotting have shown that O-GlcNAc is enriched in synapses, the specialized cell-cell junctions over which neurons communicate (Akimoto et al. 2003; Tallent et al. 2009; Cole and Hart 2001). More than 19% of all synaptic proteins are O-GlcNAcylated (Trinidad et al. 2012). In the presynaptic terminal, there is dense staining around synaptic vesicles (Akimoto et al. 2003) - the presynaptic proteins bassoon and piccolo are among the most heavily O-GlcNAcylated proteins identified (Trinidad et al. 2012). In addition to synapses, there is dense immunostaining in the nucleus in many neurons (Akimoto et al. 2003; Rex-Mathes et al. 2001; Liu et al. 2012).

On individual proteins, O-GlcNAc sites tend to cluster in disordered regions or on amino acid chain turns. A subset is found in local contexts rich in prolines (Trinidad et al. 2012; Alfaro et al. 2012; Vosseller et al. 2006; Wang et al. 2017). Both functionally and structurally, O-GlcNAc is known to cross-talk with phosphorylation (Wang et al. 2010b; Hart et al. 2011; Wang et al. 2008). In whole-cell preparations from cortex many O-GlcNAc sites are the same as or in close proximity to phosphorylation sites (Alfaro et al. 2012). Kinases in the brain seem to become modified by O-GlcNAc more frequently than other proteins (Alfaro et al. 2012; Trinidad et al. 2012). In reverse, OGT and OGA are phosphorylated (Hart et al. 2011). As discussed below, there are many instances in the brain where O-GlcNAcylation and phosphorylation regulate each other. Though, a comparison between thousands of O-GlcNAc and phosphorylation sites in synapses did not find that their sites were more often the same or closer together than what is predicted by chance (Trinidad et al. 2012).

To summarize, O-GlcNAc modifies a diverse set of proteins in the nervous system. What particular proteins become modified, even under basal conditions, depends on brain area, cell type within each area and on the age of the organism.

### The expression of OGT in the nervous system

Brain is one of the organs where OGT expression and activity are the highest (Kreppel et al. 1997; Okuyama and Marshall 2003). Overall OGT protein levels and activity *in vivo* are mostly stable during development through adulthood (Yanagisawa and Yu 2009; Liu et al. 2012). Neuronal differentiation *in vitro*, in contrast, has been associated with a small decrease in OGT mRNA and protein abundance (Andres et al. 2017; Maury et al. 2013). In the very old brain, the total protein level may go down slightly (Fulop et al. 2008). The hippocampus and the cerebellum, similar to what was described above for O-GlcNAc, are areas with particularly high OGT expression (Liu et al. 2004a; Liu et al. 2012). Biochemical fractionation has identified OGT in all major subcellular compartments of neurons (Lagerlof et al. 2017; Tallent et al. 2009). OGT is enriched in the presynaptic and postsynaptic terminals, where its specific activity is higher than in the whole-brain homogenate (Tallent et al. 2009; Lagerlof et al. 2017; Akimoto et al. 2003; Cole and Hart 2001). At least 80% of all excitatory postsynaptic terminals stain positive for OGT (Lagerlof et al. 2017). As might be expected from its broad localization pattern within neurons and the wide variety of neuronal proteins that are O-GlcNAcylated, a yeast two-hybrid screen looking for OGT-binding partners in a fetal brain cDNA library identified 27 proteins from diverse functional categories such as transcription factors, scaffolding proteins and transmembrane receptors (Cheung et al. 2008).

Through alternative splicing, at least five major transcripts coding for OGT have been described in mammals (Nolte and Muller 2002; Kreppel et al. 1997; Lubas et al. 1997; Hanover et al. 2003; Shafi et al. 2000). These are thought to give rise to three major protein isoforms, nucleocytoplasmic OGT (ncOGT), mitochondrial OGT (mOGT) and short OGT (sOGT). The isoforms differ in their N-terminal domain, which is believed to constitute the substrate for many protein-protein interactions. The full-length isoform, ncOGT, and the shortest isoform, sOGT, are present in the nucleus and cytosol. The N-terminus of mOGT includes a mitochondrial signal sequence and is present in the mitochondria (Hanover et al. 2003; Hart et al. 2011). The transcript believed to correspond to ncOGT is identified consistently in cDNA derived from brain (Lubas et al. 1997; Hanover et al. 2003; Nolte and Muller 2002; Kreppel et al. 1997; Shafi et al. 2000). Similarly, four out of five papers appear to describe the transcript corresponding to mOGT in brain (Kreppel et al. 1997; Nolte and Muller 2002; Lubas et al. 1997; Shafi et al. 2000). Whether this transcript is translated to protein has been questioned because the mOGT open reading frame in most species, including mouse but not human, includes a stop codon (Trapannone et al. 2016). From which transcript sOGT originates has not been defined definitively, but two papers that used human cDNA failed to identify in brain any transcripts other than the ones coding for ncOGT and mOGT (Lubas et al. 1997; Nolte and Muller 2002). However, transcripts that may give rise to sOGT were discovered in brain cDNA in three papers that used rat or mouse tissue (Kreppel et al. 1997; Hanover et al. 2003; Shafi et al. 2000). In whole-cell material, multiple subcellular fractions or immunoprecipitates, western blotting for OGT using several different antibodies, including antibodies known to recognize a band running at the proper size for sOGT in other tissues, typically picks up only ncOGT with certainty (Kreppel et al. 1997; Marz et al. 2006; Lagerlof et al. 2017; Okuyama and Marshall 2003; Cole and Hart 2001). Though, sOGT may become increasingly prominent with age (Liu et al. 2012). Experiments based on tools that can identify the OGT isoforms unequivocally to measure the relative amounts of ncOGT, mOGT and sOGT in the brain are needed. Notwithstanding, the current data suggest that ncOGT is highly abundant in neuronal synapses and is the major isoform present in the brain, albeit with possible species and age differences in OGT expression patterns.

### The expression of OGA in the nervous system

As for OGT, the expression and specific activity of OGA in the brain are among the highest of all organs (Dong and Hart 1994; Gao et al. 2001). Total activity appears to decrease postnatally and, *in vitro*, neuronal differentiation is associated with lower total OGA protein levels (Liu et al. 2012; Maury et al. 2013; Andres et al. 2017). There are two splice variants of OGA, full-length OGA (OGA) and short OGA (sOGA). sOGA lacks an acetyltransferase-like C-terminal domain (Butkinaree et al. 2008; Comtesse et al. 2001; Heckel et al. 1998; Toleman et al. 2004). Both isoforms are present in the brain (Comtesse et al. 2001; Heckel et al. 1998). With western blotting it was shown that a band reactive to an anti-OGA antibody migrating at the size of sOGA is downregulated at birth. The general abundance of OGA *in vivo* remains at a consistent level throughout life, apart from a peak perinatally (Liu et al. 2012). Immunohistochemistry for OGA in the adult brain resembles the staining pattern for OGT (Liu et al. 2012; Yang et al. 2017). OGT and OGA sometimes co-exist in the same complex in peripheral cells and the abovementioned yeast-two hybrid study on binding partners to OGT using brain cDNA picked up OGA (Whisenhunt et al. 2006; Slawson et al. 2008; Cheung et al. 2008). OGA is present, phosphorylated and highly active in purified synaptosomes from brain (Cole and Hart 2001; Trinidad et al. 2012). When separating the postsynaptic density (PSD) from the presynaptic fraction OGA in contrast to OGT was largely excluded from the PSD under basal conditions (Lagerlof et al. 2017). The lack of completely overlapping subcellular localization between OGT and OGA may explain in part why manipulation of OGT or OGA does not always yield the complimentary result.

### The regulation of O-GlcNAc in the nervous system

#### Complex nutrient and stress sensing

Fasting mice for 24 hours or longer leads to a strong reduction in global O-GlcNAc in the hippocampus and cortex. O-GlcNAc returns to its original levels after re-introducing food to the animals, but only after several hours (Li et al. 2006; Liu et al. 2004a). There is a delay between postprandial surges in blood glucose and the glucose concentration in cerebrospinal fluid (CSF) that is probably due to the blood-brain barrier. However, feeding increases CSF glucose within 40 minutes (Steffens et al. 1988; Silver and Erecinska 1994) and while the glucose must be metabolized to UDP-GlcNAc once it has been taken up by the cell until the rise in nutrient availability can be detected by OGT, the HBP prolongs the lag by merely a few minutes, as extrapolated from results in adipocytes (Marshall et al. 2004). The reason behind the comparatively slow restoration of hippocampal O-GlcNAc levels *in vivo* is unclear. Glucose-stimulated arrest of mitochondrial motility in dissociated cultures of hippocampal neurons was prevented by inhibiting flux through HBP. Affecting OGT or OGA function or O-GlcNAcylation of the protein Milton had the same effect, indicating that O-GlcNAcylation on some proteins in hippocampal neurons may respond *in vitro* to changes in energy over time scales measured in minutes. Exposing the axons only to glucose, by culturing the cells in a microfluidic system, appeared to hinder mitochondrial movement locally (Pekkurnaz et al. 2014). Removing leptin, an adipokine, causes hyperphagia and obesity (Williams and Elmquist 2012). Despite the surplus of available energy, Ob/Ob mice, where the gene producing leptin has been knocked out (KO), showed, in contrast, lower levels, and altered subcellular distribution of O-GlcNAc in the hippocampus, possibly due to a reduction in OGT. Caloric restriction reversed the effects on O-GlcNAc and OGT but only in some parts of the hippocampus (Jeon et al. 2016). The mechanism behind these findings is difficult to interpret because leptin may regulate O-GlcNAc directly, independently of the metabolic state of the animal (Zimmerman and Harris 2015; Harris and Apolzan 2015; Buse et al. 1997). There is much evidence showing that insulin, another metabolic hormone, phosphorylates and activates OGT through the insulin receptor (Whelan et al. 2008). Feeding animals a ketogenic diet, which may affect HBP flux, altered OGT and OGA gene expression without affecting total O-GlcNAc content in the prefrontal cortex (Newell et al. 2017). Hence, in the hippocampus, there is clear evidence that neuronal O-GlcNAc levels are nutrient-dependent. Nevertheless, the metabolic history of the animal or metabolic hormones such as leptin and insulin affect when and where O-GlcNAc is incorporated. There is no simple relationship between acute energy availability and overall O-GlcNAcylation, at least not *in vivo*.

Adding sucrose to the diet elevates global O-GlcNAc in a brain region ventral to the hippocampus, the hypothalamus (Zimmerman and Harris 2015). In the hypothalamus, however, there are cells where O-GlcNAc has been shown to be positively, negatively or (so far) not regulated by changes in energy flux. In agouti-related peptide (Agrp) neurons in the arcuate nucleus of the hypothalamus, fasting increased O-GlcNAc and OGT protein levels (Ruan et al. 2014). A similar phenomenon was discovered in cultured neuroblasts. Depriving Neuro-2a (N2a) neuroblastoma cells completely of glucose decreased O-GlcNAc after one hour but caused a marked induction after 6-9 hours. The initial drop in O-GlcNAc occurred simultaneous to higher OGA activity and reduced UDP-GlcNAc concentration. The subsequent O-GlcNAc incorporation was related to AMP-activated protein kinase (AMPK)-dependent stimulation of *Ogt* transcription (Cheung and Hart 2008), possibly as part of a stress response (Zachara et al. 2004). I will discuss below that O-GlcNAc regulates and is regulated by the response to acute cerebral insults, like stroke, and neurodegeneration occurring over months or years. There is no evidence that the O-GlcNAc response in Agrp neurons upon fasting, though, should be interpreted as a cytoprotective reaction. Ghrelin, a hormone released by the stomach in times of food deprivation, had the same effect as fasting in Agrp neurons - it increased OGT abundance (Ruan et al. 2014). It was not tested whether AMPK mediates the effect of ghrelin and fasting on OGT protein levels in Agrp neurons but it is known that ghrelin activates AMPK in the hypothalamus (Ronnett et al. 2009). The available data suggest that the induction of OGT in Agrp neurons may signal the need for preserving energy on the whole-body level (see below). In another part of the hypothalamus, in the paraventricular nucleus (PVN), fasting decreased cellular O-GlcNAc levels in αCaMKII-positive neurons but not in αCaMKII-negative neurons. This effect could be replicated *ex vivo*. Using organotypic PVN cultures, switching from 1mM to 5mM glucose for one hour increased somatic O-GlcNAc in αCaMKII-positive neurons. There was a further increase when comparing 5mM with 16 hours of 25mM glucose. Neighboring αCaMKII-negative cells, in contrast, did not react in terms of O-GlcNAc to these treatments (Lagerlof et al. 2016). These data suggest that the variability among neurons in how their O-GlcNAc levels are regulated by energy availability cannot solely be explained by elements extrinsic to the cells such as differential exposure *in vivo* to factors deriving from whole-body metabolism. Rather, the data could be explained by different intrinsic sensitivity to nutrient and hormonal access between different types of neurons. The reason behind this difference is entirely unexplored but may be partly related to alternative regulation of flux through the HBP via GFAT phosphorylation or isoform expression or the obesity-linked enzyme that can counteract GFAT, *Gnpda* (Oikari et al. 2016; Oki et al. 1999; Schleicher and Weigert 2000; Ouyang et al. 2016).

Within the same neuron, nutrient-dependent O-GlcNAcylation may not impress on all proteins equally. In the Introduction it was described that UDP-GlcNAc levels affect OGT substrate specificity and that OGT is thought to operate like a holoenzyme where its binding partners contribute to determining its targets. Removing leptin seems to alter the subcellular distribution of O-GlcNAc in the CA3 region of the hippocampus (Jeon et al. 2016). In Agrp neurons fasting increased the binding of OGT to the potassium channel Kcnq3 (Ruan et al. 2014) and glucose deprivation in N2a cells targeted OGT via the protein p38 to a different set of substrates (Cheung and Hart 2008). Under basal conditions, in neuronal cells, the protein phosphatase-1 interactor myosin phosphatase targeting subunit 1 (MYPT1) affects the substrate specificity of OGT (Cheung et al. 2008). OGT-binding proteins in brain may regulate also the specific activity of OGT, as suggested for the neurodegenerative disease protein Ataxin-10 (Marz et al. 2006).

In sum, O-GlcNAc levels have been shown to fluctuate depending on nutrient availability in neuronal cell culture, brain slice preparations and in the brain of living animals. The mechanism by which this occurs, however, is multifaceted and differs between types of neurons.

#### Neuronal activity-dependent regulation of O-GlcNAc

Particularly in some parts of the hypothalamus, physiological changes in glucose concentration promote or suppress neuronal firing (Marty et al. 2007). Neuronal firing by itself is a highly energetic event (Rangaraju et al. 2014). There is evidence nonetheless that neuronal activity regulates O-GlcNAcylation independently of indirect changes in energy consumption of the firing neuron.

Depolarizing NG-108-15 cells - a neuroblast-glioma hybrid cell type - with potassium chloride or glutamate increased global O-GlcNAc. The increase occurred within one minute, probably by direct activation of OGT (Song et al. 2008). Unlike many other glycosyltransferases utilizing a UDP-sugar, divalent cations like calcium ions are not required for OGT activity *in vitro* (Haltiwanger et al. 1990). Neither do calcium ions or ethylenediaminetetraacetic acid (EDTA) have any effect on OGA activity (Dong and Hart 1994). Rather, inhibiting voltage-dependent calcium ion influx or calcium-/calmodulin-dependent protein kinases (CaMK) blocked the effect and CaMKIV phosphorylated OGT (Song et al. 2008). Similarly, depolarization of cultured neurons induced O-GlcNAcylation of CREB at S40. The effects could be blocked by inhibiting CaMKs but also mitogen-activated protein kinase (MAPK) (Rexach et al. 2012). Raising network firing through blocking inhibitory neurotransmission in cultured primary cortical cells also was associated with an increase of O-GlcNAc on many proteins in the PSD (Lagerlof et al. 2017). These observations indicate that neuronal activity can evoke other pathways than HBP flux, such as activity-dependent phosphorylation to affect O-GlcNAcylation.

Surprisingly, the activity-dependent O-GlcNAcylation of CREB did not reach its maximum until after 6 hours (Rexach et al. 2012). Plus, inducing seizures in mice with injections of kainic acid raised O-GlcNAc on many proteins in cortex, for example on the transcription factor early growth response-1 (EGR-1), not at maximum seizure activity (two and a half hours post injection) but when the animals had started to rest (six hours post injection) (Khidekel et al. 2007). Complex modulation of neuronal activity through chemical long-term potentiation and depression, cLTP and cLTD, respectively, of hippocampal synapses in slices appear to increase global O-GlcNAc (Yang et al. 2017). In contrast, inducing LTD with low-frequency stimulation or inhibiting gamma-aminobutyric (GABA) receptors in hippocampal slices or *in vivo*, which leads to hyperexcitability, did not affect total O-GlcNAc levels in the hippocampus (Stewart et al. 2017; Taylor et al. 2014).

How neurotransmission may regulate O-GlcNAc was anticipated in an early study done in cultured, immature cerebellar neurons. Directly stimulating or inhibiting various activity-dependent kinases and phosphatases showed that while several change the O-GlcNAcylation of many proteins, the effect differs depending on substrate cohort. Inhibiting protein kinase C (PKC), for example, increased O-GlcNAc on cytoskeletal proteins but decreased O-GlcNAc on membrane and some cytosolic proteins (Griffith and Schmitz 1999; Giese and Mizuno 2013). Hence, the difficulty in identifying activity-dependent changes in overall O-GlcNAc levels may result from simultaneous up- and downregulation or subcellular-specific regulation of OGT or OGA. The conflicting results regarding whether, and under what time scale, neuronal activity directly regulates O-GlcNAcylation may be resolved by studying what signaling pathways affect OGT and OGA function in specific subcellular compartments.

#### Summary

Cross-talk between phosphorylation and O-GlcNAc, enzymatic targeting, metabolic and stress signaling are regulatory principles that apply to O-GlcNAc cycling in the nervous system, just like in peripheral cells (Hart et al. 2011; Lagerlof and Hart 2014). Nevertheless, the identity of the cell, its localization within the nervous system and its extended morphology, will modify the O-GlcNAc response to a given stimuli. Stress-, activity- and nutrient-dependent regulation of O-GlcNAc seem to occur through different pathways, but may interact as well. The activity-dependent protein kinase A (PKA) phosphorylates the two GFAT isoforms that are expressed in brain, GFAT1 and, which appears to be the main brain isoform, GFAT2. Whereas PKA can activate GFAT1, PKA inhibits GFAT2 (Hu et al. 2004; Chang et al. 2000; Oki et al. 1999). Another example of neuronal firing - HBP flux co-regulation exploits astrocytes. After the neurotransmitter glutamate has been released at synapses, it is taken up by astrocytes. Astrocytes convert glutamate to glutamine by glutamate synthetase (GS) (Schousboe et al. 2014). Glutamine, in turn, feeds into the HBP (Hart et al. 2011). Treating astrocytes with ammonia dramatically elevated global O-GlcNAc levels. The effect was not related to changes in pH or osmolarity but rather stimulation of GS and increased UDP-GlcNAc production (Karababa et al. 2014). In the behaving animal with intact neuronal circuits many factors are brought together to determine the O-GlcNAcylation of a particular protein at a particular subcellular localization in a particular cell.

## The role of O-GlcNAc in the development of the nervous system

Above it was described that the expression of the O-GlcNAc modification and its regulatory enzymes OGT and OGA change during brain development. After fluctuating during early neuronal differentiation, later development was associated with a global decrease in O-GlcNAc levels.

The developmental decrease in O-GlcNAc appears to be important for the early differentiation and organization of the nervous system. O-GlcNAc's general and essential function in cell proliferation critically affects early embryogenesis (Shafi et al. 2000; Slawson et al. 2005; Webster et al. 2009; Dehennaut et al. 2007; Dehennaut et al. 2008). O-GlcNAc may have additional effects on stem cell renewal and differentiation. Several transcription factors that regulate pluripotency - such as Octamer-binding protein 4 (Oct4) - are O-GlcNAcylated in stem cells. There are several O-GlcNAc sites on Oct4 and its transcriptional activity is increased by overexpression of OGT. Upon differentiation, the O-GlcNAc modification is lost (Jang et al. 2012). Mutating the O-GlcNAc site T228 to alanine on Oct4 repressed its transcriptional activity and the renewal of mouse embryonic stem cell colonies. Partly due to other O-GlcNAc sites, the role of O-GlcNAc for Oct4 and stem cell biology is however multifaceted (Jang et al. 2012; Constable et al. 2017; Miura and Nishihara 2016). Whereas the effect may differ depending on culturing protocol, inhibiting OGT or OGA pharmacologically *in vitro* has been shown not to affect human stem cell pluripotency, as measured by the expression of, e.g., Oct4 (Andres et al. 2017; Maury et al. 2013; Speakman et al. 2014). Rather, globally decreasing or increasing O-GlcNAc levels respectively accelerated or delayed the commitment to a neuronal fate (Andres et al. 2017; Maury et al. 2013). Stimulating the differentiation of orexin neurons, a cell type regulating the sleep/wake cycle and feeding behavior, is associated with a switch from OGT to OGA occupancy in the *Orexin* gene promotor region. OGA activity increases and OGT decreases orexin expression (Hayakawa et al. 2013). Surprisingly, deletion of OGA in all tissues *in vivo* may lead to a general developmental retardation but no gross intrauterine histological defects (Yang et al. 2012; Keembiyehetty et al. 2015). In contrast, altering global O-GlcNAc levels in Zebrafish embryos in either direction was associated with cell death and dramatic changes in brain structure. Only increased O-GlcNAc levels, however, disrupted the gross patterning of the central nervous system (Webster et al. 2009). Removing OGA in mice from ectoderm, the tissue lineage giving rise to the nervous system, leads to severe developmental perturbation in several brain regions such as cortex, the hippocampus and the pituitary. The number of dividing and neuronal precursor cells was elevated, but loss of OGA suppressed progression to mature neurons *in vivo* and *in vitro* (Olivier-Van Stichelen et al. 2017).

Hyperglycemia is linked to several developmental defects, including failure of the neural tube to close (Tan et al. 2017b). Neural tube closure is regulated by Paired box 3 (PAX3), a transcription factor believed to cause many cases of the developmental condition called the Waardenburg syndrome. PAX3, or a PAX3-binding protein, has been found to be O-GlcNAcylated. Raising O-GlcNAc levels by injecting glucose or the highly specific OGA inhibitor Thiamet G (TMG) into chicken egg embryos decreased PAX3 protein expression, while hyperglycemia did not affect PAX3 mRNA levels. In reverse, the glucose-induced loss of PAX3 could be rescued by blocking OGT and O-glycosylation in the Golgi apparatus with benzyl-2-acetamido-2-deoxy-α-D-galactopyranoside (BG) (Tan et al. 2017b). Applying the putative OGT inhibitor ST045849 to pregnant diabetic mice similarly decreased the number of pups suffering from neural tube closure defects (Kim et al. 2017). Hence, another mechanism by which O-GlcNAc regulates the early development of the nervous system may be through inducing PAX3 degradation and thus affect neural tube closure.

Culturing primary neurons has indicated that later neuronal maturation depends on relatively low O-GlcNAc levels. Overexpressing OGA stimulates neuronal polarization, increases the density of filopodia on axons and the number of neurons displaying branched processes. Inhibiting OGA pharmacologically decreased the number of axonal protrusions, probably at least in part by inhibiting PKA (Francisco et al. 2009). In another study, OGT overexpression decreased and OGT KO increased axon length. Knocking down CREB, which is known to regulate axon growth, prevented the effect of OGT overexpression or KO (Rexach et al. 2012). OGT has been shown to stimulate PKA activity and phosphorylation of the activating PKA site S133 on CREB (Xie et al. 2016). However, the OGT-dependent effect on axons was related to direct O-GlcNAcylation of CREB on S40. S40 O-GlcNAcylation is induced by neuronal depolarization, as discussed above, but only after S133 has become phosphorylated. O-GlcNAc appears to turn off further CREB-dependent transcription by disrupting the binding of CREB to its co-activator CREB-regulated transcription coactivator (CRTC). O-GlcNAcylation of S40 regulated dendritic growth as well. The effect on dendritic *versus* axonal elongation was mediated by different downstream CREB gene products; Wnt2 for dendrites and BDNF for axons (Rexach et al. 2012). O-GlcNAcylation of CREB may inhibit activity-independent transcription also by perturbing the complex between CREB and TAFII130, a component of the TFIID transcriptional complex, and total CREB protein levels (Lamarre-Vincent and Hsieh-Wilson 2003; Rexach et al. 2012; Xie et al. 2016).

Establishing neuronal networks relies at the end on neurons forming synapses. The neuronal networks present when development has run its course should not however be conceived of as finished products. The number and strength of neuronal synapses continue to be molded in adulthood. So-called synaptic plasticity too is affected by O-GlcNAc; O-GlcNAc's role in these processes will be discussed below.

O-GlcNAc cycling regulates numerous steps throughout the development of the nervous system. A common theme in the central nervous system appears to be that decreasing O-GlcNAc promotes, while increasing O-GlcNAc inhibits neuronal differentiation, extension and organization. Though, knocking out OGT specifically in neurons, thereby decreasing O-GlcNAc dramatically, lead to fewer KO pups born than expected and those animals that survived until term never developed locomotor behavior but died within ten days (O'Donnell et al. 2004). Culturing neurons dissociated from dorsal root ganglia in the peripheral nervous system of four-week-old mice demonstrated in addition that deleting OGT reduced axon growth (Su and Schwarz 2017). At least three different mutations in *Ogt* have been discovered to segregate in humans with severe intellectual disability. The mutations were associated with lower OGT protein levels. Nonetheless, global steady-state O-GlcNAc did not change, possibly due to compensation in OGA. How disease results from these mutations is unclear but instead of altered total O-GlcNAc, the underlying explanation may be altered O-GlcNAc cycling kinetics or substrate targeting of OGT; the mutations lie in the TPR-domain of OGT which mediates many protein-protein interactions (Willems et al. 2017; Vaidyanathan et al. 2017). The role of O-GlcNAc during development has only begun to be investigated. Once the molecular mechanisms are dissected further, other principles than overall fluctuations may arise. Global and pathway-specific rules of O-GlcNAc functioning are bound to intersect to assure proper development of the peripheral and central nervous system.

## The regulation of animal behavior and neuronal function by O-GlcNAc

The previous sections have showed that O-GlcNAc cycling in the nervous system occurs on proteins in many functional categories and is regulated by many stimuli that differ depending on the identity of the cell. The roles played by O-GlcNAc in the brain and the peripheral nervous system are bound to be equally diverse. Here the main functions in mature neurons and adult animal behavior that have been addressed in previous research are discussed.

### Animal behavior

#### Metabolism

Much evidence holds that the brain is a master regulator of whole-body metabolism. The brain keeps track of energy storage levels, detects acute fluctuations in nutrient supply and directs feeding behavior, energy expenditure and other components of metabolism. This control is shared by very many areas within the brain. Nevertheless, some neuronal circuits are thought to be more directly involved. The function of such metabolic circuits relies on information about the body's metabolic status they receive from hormonal, nutrient and neuronal signals from peripheral organs (Schwartz et al. 2000; Blouet and Schwartz 2010; Sohn et al. 2013; Rossi and Stuber 2018). Recent observations suggest that O-GlcNAc is integral to the way body-to-brain signals are sensed by the retrieving neuron and integrated into its larger network.

Many genes linked to obesity are believed to act in the brain (Locke et al. 2015). One of the most common obesity genes, *Gnpda2*, regulates flux through the HBP (Speliotes et al. 2010; Gutierrez-Aguilar et al. 2012; Wolosker et al. 1998). The gene for *Oga* is linked to late onset diabetes in Mexican Americans and possibly spontaneously occurring diabetes in so-called Goto-Kakizaki rats (Duggirala et al. 1999; Lehman et al. 2005; Keembiyehetty et al. 2015; Xue et al. 2015). The effects of removing OGA in mice during embryogenesis on brain development have differed markedly depending on how the mouse line was established. Results show consistently, however, that OGA KO homozygotes are born with lower body weight and die within a few days. The cause of death has been associated with either pulmonary malfunction or hypoglycemia (Keembiyehetty et al. 2015; Yang et al. 2012). Heterozygotes, on the other hand, survive development and are fertile (Yang et al. 2015; Keembiyehetty et al. 2015). Total food intake is not disturbed in these mice but the respiratory exchange ratio (RER) is increased, suggesting heightened preference for utilizing carbohydrates instead of fatty acids in energy expenditure. One group has demonstrated increased overall energy expenditure, improved glucose tolerance and lower body weight due to a more lean body type. Their mice were partly resistant also against obesity when fed a high-fat diet (HFD) (Yang et al. 2015). Heterozygote females from another line of OGA KO mice, in contrast, were heavier than controls on either regular chow or HFD, whereas there was no change in body weight or composition for males (Keembiyehetty et al. 2015). Systemic application of OGA inhibitors over weeks in adult mice and rats has been reported to not alter total food intake, body weight or glucohomeostasis (Yuzwa et al. 2012; Macauley et al. 2010).

It has been speculated that the phenotype of the whole-body deletion of OGA derives from adipose tissue, but these studies did not rule out other organs (Yang et al. 2015). Removing in mice OGA selectively in ectoderm, which includes neurons, does not cause premature death or affect fertility. Without affecting overall body weight or food intake, adipose levels were increased (Olivier-Van Stichelen et al. 2017). Neuronal KO of OGT during development leads to lower body weight but the effect on locomotor behavior is so severe that no straightforward argument can be made for a direct effect on metabolic regulation (O'Donnell et al. 2004). A thorough metabolic analysis of the known OGT mutations in human subjects has not been performed (Willems et al. 2017; Vaidyanathan et al. 2017).

Systemic and brain-wide perturbations suggest that neuronal O-GlcNAc regulates metabolism. But the data are conflicting as to what may be the underlying mechanism. Instead, targeting OGT mutations to specific neurons in the hypothalamus - a brain region known to influence metabolic behavior directly - indicates that O-GlcNAc plays different roles in different types of neurons.

Agrp neurons in the hypothalamus become activated upon fasting and are associated classically with the control of foraging behavior and food intake in adult animals (Williams and Elmquist 2012; Aponte et al. 2011; Sohn et al. 2013). Ablating Agrp neurons during development does not lead to any major effects on food intake, probably due to compensation from other pathways (Luquet et al. 2005; Williams and Elmquist 2012). The Agrp neurons also regulate energy expenditure and glucose homeostasis (Wang et al. 2014b; Small et al. 2001). It was recently reported that Agrp neurons upon fasting decrease energy expenditure at least in part by inhibiting the thermogenic effect (TE) in retroperitoneal white fat (rWAT), so-called browning (Ruan et al. 2014). Upon deleting OGT from Agrp neurons during development, there were no discussed effects on cellular or animal viability. Whole-cell patch clamp did not show any difference in membrane potential between OGT KO and Wt cells. There was neither any change in daily food intake or body weight in freely behaving mice, but glucose tolerance was improved and energy expenditure decreased less than in Wts upon subjecting the mice to a fast. Loss of OGT made the mice partly resistant to detrimental effects of HFD. As described above, fasting induces OGT and O-GlcNAc abundance in Agrp neurons. OGT removal decreased the Agrp neuron firing frequency, possibly through modifying the Kcnq3 potassium channel at T665. It seems that in Agrp neurons OGT prevents energy expenditure during periods of caloric restriction (Ruan et al. 2014).

In adult mice, deleting OGT in αCaMKII neurons increased body weight rapidly. Daily food intake more than doubled. There was no change in meal frequency but KO animals ate larger and longer meals. Energy expenditure in mice fed *ad libitum* was elevated also, at least to some extent due to higher physical activity. Allowing the OGT KO mice to eat only as much as Wts blocked any change in body weight. Removing OGT locally from αCaMKII neurons in the PVN in adult mice by stereotactic virus injection caused hyperphagia and obesity as well. Fasting decreased and glucose increased O-GlcNAc abundance in the αCaMKII PVN neurons, as discussed above. It has been shown that removing OGT from αCaMKII neurons during early postnatal development leads to cellular degeneration of αCaMKII neurons in the hippocampus or cortex (Wang et al. 2016). Cell number in the PVN, or in the hippocampus, was not affected by acute deletion of OGT in adults, and just like in Agrp cells there was no effect on membrane potential (Lagerlof et al. 2016). Glutamatergic neurotransmission in the PVN has been shown to inhibit feeding (Hettes et al. 2003; Fenselau et al. 2017). Loss of OGT was associated with a decrease in excitatory synapses and blocked feeding-induced activation of the αCaMKII PVN neurons, as measured by immunohistochemistry for the immediate-early gene cfos (Lagerlof et al. 2016; Lagerlof et al. 2017). Optogenetic stimulation of these cells decreased food intake and meal size. These data suggest that OGT in adult animals couples food intake with caloric need at least in part by its regulation of excitatory synapses in αCaMKII PVN neurons (Lagerlof et al. 2016).

Thus, there are many indications that energy availability regulates O-GlcNAc signaling in the brain and that O-GlcNAc cycling in neurons affects whole-body metabolism. The precise role played by O-GlcNAc, however, depends on the type of cell and the age of the animal.

#### Learning and memory

Animal behavior relies on processes where information about previous experience is used to overcome challenges like avoiding predators and locating food (Bailey and Kandel 1993; Bhatt et al. 2009; Verpelli and Sala 2012; Grant 2012). Many forms of learning and memory depend on the hippocampus (Lynch 2004). Two such behaviors are novel object and placement recognition (NOR and NOP, respectively) (Taylor et al. 2014; Antunes and Biala 2012; Vogel-Ciernia and Wood 2014). The animal is habituated initially to two objects and then, when put back in the same arena two hours later, tested for its ability to recognize that one of the objects or its location has been switched. Injecting TMG systemically prior to habituation impaired the performance in NOR and NOP, indicating that memory acquisition or retrieval were compromised (Taylor et al. 2014). In a different hippocampal-dependent spatial learning task, both learning and memory were interpreted as diminished in OGA KO heterozygotes (Yang et al. 2017). Contextual fear conditioning (CFC) pairs a new environment with foot shocks and measures the degree of freezing in the same environment 24 hours later. CFC relies on the hippocampus but also brain areas such as the amygdala. Injection of TMG prior to conditioning did not affect the fear response 24 hours later (Taylor et al. 2014). In contrast, OGA KO heterozygotes exhibited markedly decreased freezing in a similar CFC task (Yang et al. 2017). Another group lowered global O-GlcNAc in the brain by injecting 6-diazo-5-oxo-norleucine (DON), a glutamine analog that inhibits glutamine-utilizing pathways like the HBP and many others (Hart et al. 2011), into the cerebral ventricles prior to a contextual and cued FC task by which the learning and memory components can be addressed, to some extent, separately. DON impaired both the learning and memory retrieval phases of the experiment (Xie et al. 2016). As there were no effects in the open-field or the rotarod tests, the behavior in these tasks from globally perturbing O-GlcNAc levels genetically or pharmacologically cannot be explained by generally detrimental effects on motor coordination or exploratory behavior (Taylor et al. 2014; Yang et al. 2017). Fear conditioning has been described to increase O-GlcNAcylation on CREB. Above it was discussed that mutating the O-GlcNAc site S40 on CREB to alanine (S40A) increases the transcription of several genes known to affect learning and memory, e.g. *Bdnf*. Injecting S40A CREB into the amygdala, as compared to injecting Wt CREB, enhanced freezing in FC two hours after training, suggesting that inhibiting CREB O-GlcNAcylation improved memory formation (Rexach et al. 2012).

Pharmacological, genetic and site-specific data indicate that several short- and long-term learning and memory behaviors rely on O-GlcNAc cycling. In reverse, as discussed above, perturbed O-GlcNAcylation is associated with intellectual disability in humans (Vaidyanathan et al. 2017; Willems et al. 2017). Below will show that the mechanism underlying this phenotype is probably complex and requires interrogation of discrete pathways in specific cell-types.

#### The peripheral nervous system

The peripheral nervous system lies mainly outside the blood-brain barrier. Here, cells are likely to experience larger fluctuations in energy availability and to some extent different metabolic hormonal signaling (Hoyda et al. 2009; Su and Schwarz 2017). In mice, removing OGT during early development from Schwann cells, the glia that myelinate peripheral nerves, lead to several behavioral stigma associated with neuromuscular dysfunction such as muscle weakness and gait abnormalities at six months of age. Electrophysiological investigations verified that the function of motor and sensory neurons was severely defective. While the number of Schwann cells had not changed, there was a clear and progressive loss of axons. Demyelination occurred, but the axonal degeneration started prior to that. O-GlcNAc mapping of proteins in sciatic nerve tissue identified among 122 others a myelin protein called periaxin (PRX) which is known in humans to be associated with neuropathy. PRX was mislocalized in OGT KOs and may explain in part how OGT in Schwann cells regulates myelin homeostasis and supports axon integrity (Kim et al. 2016). Knocking out OGT in peripheral sensory neurons directly caused striking hyposensitivity to mechanical and thermal stimulation. Regardless if OGT was deleted during development or in adults, the deletion was associated with cell loss, suggesting that OGT is essential for sensory neuron maintenance (Su and Schwarz 2017).

### The regulation of synapses and neuronal signal transmission

#### Excitatory synapses and AMPA receptor trafficking

It is believed that the brain learns and establishes new memories through modulating the number or strength of synapses, so-called synaptic plasticity (Bailey and Kandel 1993; Bhatt et al. 2009; Zuo et al. 2005a; Jontes and Phillips 2006; Shepherd and Huganir 2007). Much research has focused on how excitatory synapses in the hippocampus encode experience. The majority of fast excitatory neurotransmission in the brain is conducted by α-amino-3-hydroxy-5-methyl-4-isoxazolepropionic acid (AMPA) receptors. The AMPA receptor is a tetrameric glutamate receptor composed of four subunits, GluA1-4. In forebrain neurons, two major isoforms are GluA1/2 and GluA2/3 (Shepherd and Huganir 2007). At least in the adult cortex and hippocampus, most excitatory synapses occur on dendritic protrusions called spines. AMPA receptors trafficking in or out of synapses while spines enlarge and stabilize or become thinner and retract leading to synaptic LTP or LTD is believed to constitute the substrate of many memory procesess (Collingridge et al. 2004; Sala and Segal 2014; Bailey and Kandel 1993; Bhatt et al. 2009; Jontes and Phillips 2006; Zuo et al. 2005b). Several lines of evidence show that dynamic properties of excitatory synapse biology including AMPA receptor-dependent synaptic plasticity depend on O-GlcNAc cycling.

Insertion of AMPA receptors into the synaptic cleft contributes to high-frequency stimulated (HFS) LTP of hippocampal synapses in the CA1 region (Collingridge et al. 2004). HFS LTP diminished after acutely elevating O-GlcNAc levels by either inhibiting OGA with TMG or stimulating the production of UDP-GlcNAc with glucosamine (GlcN) (Taylor et al. 2014). Similarly, hippocampal HFS LTP was decreased in OGA KO hetereozygotes (Yang et al. 2017). Several hours after another inhibitor of OGA, 9d, had been injected systemically *in vivo*, however, the HFS LTP in the hippocampus increased (Tallent et al. 2009). Attempting to inhibit OGT using the drug Alloxan has shown both enhanced and diminished LTP (Kanno et al. 2010; Tallent et al. 2009). Whether the Alloxan-dependent observations are due to perturbations of O-GlcNAcylation is questionable, though, because Alloxan is well-known for its many and unspecific targeting (Taylor et al. 2014). The induction of LTD with low-frequency stimulation (LFS), a protocol associated with the removal of AMPA receptors from synapses, was destabilized in a situation where O-GlcNAc levels had been elevated for a long period of time by deleting one copy of *Oga* during development (Yang et al. 2017; Collingridge et al. 2004). While these experiments are contradictory as to the exact timing and direction of regulation, they strongly indicate that O-GlcNAc affects dynamic synaptic properties.

The data on whether and how O-GlcNAc regulates basal synaptic properties are more tentative. There was no change in basal synaptic transmission in the hippocampus five hours after systemic injection *in vivo* of 9d (Tallent et al. 2009). Neither did permanently elevated O-GlcNAc levels upon genetically removing *Oga* yield any change in basal synaptic properties or spine number in hippocampal cells (Yang et al. 2017). Another study showed in contrast that acutely applying TMG directly to hippocampal slices leads to an almost immediate depression of synaptic strength. Raising O-GlcNAc using GlcN had the same effect. The depression persisted for at least 60 minutes and continued after global O-GlcNAc levels had returned to normal. The concept of a transient increase in O-GlcNAc leading to a long-lasting synaptic depression was dubbed O-GlcNAc LTD (Taylor et al. 2014). A separate report observed that simultaneous short-term administration of TMG and GlcN dampened also induced hyperexcitability in slices and seizure activity *in vivo* (Stewart et al. 2017). It has been speculated that O-GlcNAc LTD results from the actual O-GlcNAc increase, rather than altered absolute O-GlcNAc levels (Taylor et al. 2014).

The O-GlcNAc LTD was completely blunted in GluA2 KO animals, suggesting a shared mechanism between O-GlcNAc and LFS LTD. Notwithstanding, blocking some pathways that have been associated with classical AMPA receptor-related LFS LTD like N-methyl-D-aspartate (NMDA) receptors and PKC did not inhibit the O-GlcNAc-induced LTD. Plus, saturating O-GlcNAc LTD with repeated application of GlcN did not occlude LFS LTD. Repeated LFS did, though, block further TMG-dependent LTD (Taylor et al. 2014). Global or sparse genetic deletion of OGT in primary cultured neurons reduced the surface expression of GluA2 and GluA3 and the synaptic expression of GluA3. There was no significant decrease in GluA1 (Lagerlof et al. 2017). OGT co-immunoprecipitates with GluA2, but not GluA1, and may modify GluA2 directly (Taylor et al. 2014). Deleting OGT lead to fewer and immature spines and lower synapse number, as determined by immunohistochemistry (Lagerlof et al. 2017). These data concord with experiments *in vivo* where the frequency and amplitude of excitatory synaptic inputs on αCaMKII PVN neurons upon KO of OGT in adult mice diminished (Lagerlof et al. 2016). While it was not tested whether the effects on spines and AMPA receptors are mediated by the same mechanism, the data suggest in all that O-GlcNAc regulates the synaptic abundance of the GluA2/3 AMPA receptor isoform. Withal, cLTP and cLTD in hippocampal slices from OGA KO heterozygotes was associated with impaired phosphorylation of sites on GluA1 known to affect GluA1/2 trafficking (Yang et al. 2017).

Many proteins that regulate AMPA receptor trafficking and spine dynamics are modified by O-GlcNAc, including in synapses (Trinidad et al. 2012; Alfaro et al. 2012). αCaMKII, for example, is O-GlcNAcylated at T306 (Trinidad et al. 2012). Phosphorylation of T305 and/or T306 inhibits αCaMKII and impairs HFS LTP and learning (Elgersma et al. 2002). TMG has been shown to increase αCaMKII phosphorylation at T286/7, which activates αCaMKII (Tallent et al. 2009). Phosphorylation of S831 on GluA1, a known αCaMKII site, was blocked in a cLTP protocol in OGA KO mice (Yang et al. 2017; Shepherd and Huganir 2007). Whether O-GlcNAcylation of αCaMKII mediates any of the effects on synaptic plasticity or animal behavior associated with global alterations of O-GlcNAc levels has not been tested. Another kinase that has been reported to be O-GlcNAcylated and involved in the regulation of GluA1 trafficking and experience-dependent behavior is PKA (Kessels and Malinow 2009; Xie et al. 2016). Modulation of OGT or OGA pharmacologically or by overexpression affects PKA function consistently, but different papers report in opposite directions (Xie et al. 2016; Francisco et al. 2009).

Much data suggest that O-GlcNAcylation affects many aspects of AMPA receptor trafficking and excitatory postsynaptic function. Whereas manipulations of global O-GlcNAc levels have opened up the field, teasing apart the underlying mechanism(s) will require investigations of specific signaling pathways.

#### Presynaptic and other functions regulated by O-GlcNAc

It was described above that O-GlcNAc is highly abundant in postsynaptic terminals but also in in presynaptic terminals. In presynaptic terminals, synapsin 1 binds to synaptic vesicles and regulates synapse number and function (Skorobogatko et al. 2014). At least 16 O-GlcNAc sites have been identified on synapsin 1 (Vosseller et al. 2006; Trinidad et al. 2012; Skorobogatko et al. 2014). Mutating the T87 O-GlcNAc site to alanine in cultured primary neurons enhanced the targeting of synapsin 1 to synapses, enlarged the size of the synaptic vesicle reserve pool and increased synapse density (Skorobogatko et al. 2014). Though, global manipulations of O-GlcNAc levels by inhibiting or deleting OGA have had both no effect and a decreased presynaptic release probability, as measured by electrophysiology in the hippocampus (Tallent et al. 2009; Taylor et al. 2014; Yang et al. 2017).

In addition to direct effects on synaptic neurotransmission, O-GlcNAc may influence synapse function and neuronal transmission by modulating activity-dependent gene transcription (Rexach et al. 2012; Song et al. 2008; Dias et al. 2009). We have also seen that OGT critically regulates the spontaneous firing frequency of Agrp neurons in the hypothalamus, at least in part due to its modulation of Kcnq3 (Ruan et al. 2014). In comparison, intrinsic excitability of hippocampal neurons did not differ between wildtype and OGA KO heterozygotes (Yang et al. 2017). Also, through the protein Milton as described above, O-GlcNAc may link the high energy demand of neuronal transmission to mitochondrial movement and ATP production (Rangaraju et al. 2014; Tan et al. 2014; Pekkurnaz et al. 2014).

### Summary

O-GlcNAc has many functions in the nervous system. Depending on the cell, the regulation of O-GlcNAc cycling and the effects of increased or decreased O-GlcNAcylation are different. Between circuits, there are also shared mechanisms. Effects on synaptic plasticity, for example, may underlie both hippocampal-dependent memory behavior and hypothalamic feeding behavior. The current literature abounds with conflicting results. Some conflicts likely depend on factors such as type of preparation and length of O-GlcNAc perturbation. The most fundamental questions remain unanswered. How does the energy-sensing properties of O-GlcNAc relate to its activity-dependent regulation? At very low energy levels, as discussed above and below O-GlcNAc helps neurons to avoid cytotoxicity. However, O-GlcNAc also fluctuates in response to mild and physiological changes in energy availability. In the hypothalamus, the data favor the interpretation that O-GlcNAc detects body-to-brain metabolic signaling to regulate energy expenditure and food intake. But what role does nutrient-dependent O-GlcNAcylation play in the hippocampus? Is O-GlcNAc a mechanism by which our diet affects memory performance? Future studies linking molecular mechanisms to the function of specific neuronal circuits will uncover important concepts not only for the field of O-GlcNAc but for the field of neuroscience.

## The role of O-GlcNAc in aging, neurodegenerative disorders and acute cerebral insults

For more than two decades, O-GlcNAc has been known to modify proteins involved in the pathology behind aging-related diseases (Griffith et al. 1995; Griffith and Schmitz 1995). Today, most pathways involved in neurodegenerative disorders have been shown to contain O-GlcNAc (Banerjee et al. 2016; Hart et al. 2011; Trinidad et al. 2012; Wang et al. 2017; Alfaro et al. 2012; Lagerlof and Hart 2014). O-GlcNAcylation levels are changed in many cases of Alzheimer's and Parkinson's diseases (Forster et al. 2014; Liu et al. 2004a; Griffith and Schmitz 1995; Wani et al. 2017). OGA is linked genetically and through alternative splicing to Alzheimer's disease (Bertram et al. 2000; Heckel et al. 1998; Twine et al. 2011). The gene for OGT is associated with parkinsonian dystonia (Mazars et al. 2010; Nolte and Muller 2002; Muller et al. 1998). Metabolic disease is a risk factor for developing dementia, and symptom progression in dementia has been slowed with drugs potentiating insulin signaling in rodents and in humans (Gudala et al. 2013; Moreira 2012; Bobsin and Kreienkamp 2015; Cooper et al. 2015; Bomfim et al. 2012; De Felice et al. 2009; Escribano et al. 2010; Querfurth and LaFerla 2010). Coupling between energy availability, neuronal function and cell stress management makes O-GlcNAc an etiologic candidate for many neurodegenerative disorders. Several reviews on the connection between O-GlcNAc, aging and aging-related diseases have been published recently (Hart et al. 2011; Zhu et al. 2014; Bond and Hanover 2013; Banerjee et al. 2016; Yuzwa and Vocadlo 2014; Ma et al. 2017). Here, only larger themes will be discussed.

Histopathological hallmarks of Alzheimer's disease, a common cause of dementia, are neurofibrillary tangles and amyloid-β (Aβ) plaques. Tangles consist of aggregates of the protein tau (Masters et al. 2015). Tau is multiply modified by O-GlcNAc (Arnold et al. 1996; Yuzwa et al. 2011). O-GlcNAc levels correlate negatively with tau phosphorylation (Liu et al. 2004a; Li et al. 2006). Either by a direct effect on solubility or by protecting against hyperphosphorylation, or both, O-GlcNAcylation of tau inhibits tau aggregation (Graham et al. 2014; O'Donnell et al. 2004; Yuzwa et al. 2008; Smet-Nocca et al. 2011; Yuzwa et al. 2014a; Zhu et al. 2014; Hastings et al. 2017). Increased O-GlcNAc protects against Aβ plaque formation also (Yuzwa et al. 2014b; Kim et al. 2013). O-GlcNAc has been suggested to regulate the cleavage of amyloid precursor protein (APP), which produces Aβ or non-pathological species, and to be associated with downstream effects or the removal of Aβ (Yuzwa et al. 2014b; Kim et al. 2013; Griffith et al. 1995; Jacobsen and Iverfeldt 2011; Cha et al. 2015; Wang et al. 2012; Zhang et al. 2003; Chun et al. 2017). In mouse models where mutated tau or Aβ is overexpressed, pharmacological inhibition of OGA alleviates neuronal degeneration and improves behavioral outcome (Borghgraef et al. 2013; Yuzwa et al. 2012; Graham et al. 2014; Yuzwa et al. 2014b; Kim et al. 2013).

Parkinson's disease and Huntington's chorea are other neurodegenerative disorders distinguished by accumulation of toxic protein aggregates (Banerjee et al. 2016). Similar to the effect on tau, O-GlcNAcylation at T72 on α-synuclein, a protein associated with parkinsonian inclusion bodies, prevents its aggregation and toxicity when added to cells exogenously (Marotta et al. 2012; Marotta et al. 2015; Wang et al. 2010a). Based on these and other data, it has been suggested that O-GlcNAc may serve a general function of impeding protein aggregation (Yuzwa et al. 2012). In Huntington's chorea, expansion of CAG repeats in the gene coding for huntingtin leads to the production of a polyglutamine mutant protein that aggregates in the cell. TMG was shown to improve cell viability upon overexpressing mutant huntingtin in primary cultured neurons, but possibly via rescuing nucleocytoplasmic transport (Grima et al. 2017). In contrast to the situation in Parkinson's and Alzheimer's disease, decreasing global O-GlcNAc was shown to be protective against overexpression of huntingtin aggregation and cytotoxicity in neuroblastoma cells and in flies (Kumar et al. 2014). In fact, expressing toxic species of huntingtin, tau or Aβ in worms was associated with improved or exacerbated outcome upon removing OGT or mutating OGA, respectively (Wang et al. 2012). Inhibition of autophagy or the proteasome may, to some extent, explain the aggravating effect of increasing O-GlcNAc levels (Wang et al. 2012; Kumar et al. 2014).

In peripheral tissues, O-GlcNAc has been characterized as a general stress sensor and regulator of reactive oxygen species (ROS) (Hart et al. 2011; Tan et al. 2017a). Inducing stroke by stopping blood flow to specific areas of the brain leads to a rapid increase in total O-GlcNAc in rodents, including in the stroke penumbra zone. The penumbra zone is important clinically as it contains cells that are damaged but that are possible to rescue from cell death (Jiang et al. 2017; Gu et al. 2017). Infarction size is smaller upon systemic or intraventricular injection of TMG or GlcN. The behavioral deficits e.g. motor function, are alleviated upon global elevation of O-GlcNAc as well (Gu et al. 2017; Jiang et al. 2017; He et al. 2017). Inhibition of the HBP has the opposite effect (Gu et al. 2017). A very high dose of TMG, however, aggravated stroke outcome (Gu et al. 2017). An older study shows that intraventricular injection of streptozotocin leads to apoptosis in the hippocampus (Liu et al. 2004b). Streptozotocin blocks OGA activity but may have additional targets (Roos et al. 1998; Pathak et al. 2008). Primary cultured neurons that were treated with TMG for seven days increased monomeric α-synuclein levels and were less viable (Wani et al. 2017). In aged mice, the O-GlcNAc increase upon transient ischemia was absent (Liu et al. 2016).

O-GlcNAcylation plays a variety of roles that can be either protective or harmful to conditions associated with neuronal degeneration or acute insults. The result of global elevation or depression of O-GlcNAc differs depending on the precipitating stimuli and the timing of the event. It is also cell-specific. OGT is not necessary for Agrp neuron or Schwann cell maintenance (Kim et al. 2016; Ruan et al. 2014). But above it was described that removing OGT from sensory neurons in the peripheral nervous system was associated with neuronal death, possibly from an axonal dieback mechanism (Su and Schwarz 2017). In αCaMKII neurons, OGT is essential during early postnatal development but not in young adulthood (Wang et al. 2016; Lagerlof et al. 2016).

The systemic or brain-wide application of the drugs that have been used to manipulate O-GlcNAc affect neurons and glia. The involvement of glia in stress reactions in the brain has been recognized for decades and is becoming increasingly discussed as a potential drug target. The function of O-GlcNAc in these cells is almost entirely unknown but the protective effect of increased O-GlcNAc may to some extent relate to modulation of the inflammatory response (Salter and Stevens 2017; van den Hoogen et al. 2017; Mizuma and Yenari 2017; Ma et al. 2017; He et al. 2017). In fact, the connection between O-GlcNAc and immune cells in the periphery was identified in the very first paper on O-GlcNAc (Torres and Hart 1984). There is need for further mechanistic understanding of how O-GlcNAc on the one hand coordinates several disease-related processes within specific cells simultaneously, while, on the other hand, may respond to and regulate communication between different types of cells in the nervous system.

## Concluding remarks and outlook

The regulation and function of O-GlcNAc in the nervous system are cell-specific. In Agrp neurons, fasting seems to stimulate O-GlcNAc to inhibit energy expenditure. O-GlcNAc levels decrease in αCaMKII PVN neurons upon fasting. Loss of OGT in these cells lead to feeding-induced obesity. Many other studies indicate that O-GlcNAc in additional brain areas affects behaviors that go beyond metabolism, like learning and memory. The O-GlcNAc field has been hampered since its inception by the difficulty of identifying and studying the role of individual O-GlcNAc sites. Major efforts have lead to advances in mass spectrometry that have enabled recently the mapping of thousands of O-GlcNAc sites in samples derived from whole-brain material. The development of specific inhibitors of OGA and work *in vitro* and in neuronal cell lines have been fundamental for the understanding of O-GlcNAcylation in the brain. Drugs like TMG may prove also to have important clinical use. With models based on conditional deletion of OGT and OGA, it is becoming possible to interrogate the role of O-GlcNAc in intact and defined neuronal circuits. The widespread effects of manipulating OGT or OGA in the whole cell warrant the search for easy-to-use tools to, ideally, probe the regulation and function of specific O-GlcNAc sites in specific cells. Today we lack these tools but the fascinating biology of O-GlcNAcylation is attracting more and more scientists. The hope for discovering causal mechanisms of O-GlcNAc function may be realized sooner than one may think. The field of O-GlcNAc is bound to uncover important concepts of how the brain works in health and disease.
